# ‘EMERALD’ online early intervention programme for psychological well-being: A detailed description using the TIDieR checklist

**DOI:** 10.1177/20552076241288381

**Published:** 2024-10-15

**Authors:** Monique Jones, Alexandra P Metse, Andrew Watkins, Daniel F Hermens, Christina Driver

**Affiliations:** 1 Thompson Institute & National PTSD Research Centre, University of the Sunshine Coast, Birtinya, QLD, Australia; 2 School of Health, University of the Sunshine Coast, Sippy Downs, QLD, Australia; 3 School of Psychological Sciences, 5982 University of Newcastle, Callaghan, NSW, Australia; 4 Mindgardens Neuroscience Network, Sydney, NSW, Australia

**Keywords:** Mental health, well-being, lifestyle medicine, solution focused, health coaching, primary health care, telehealth

## Abstract

**Objective:**

The rising prevalence of mental health symptoms brought on by the COVID19 pandemic led to the inception and development of EMERging Anxiety, Loneliness, Depression (EMERALD) well-being programme. EMERALD was designed to improve psychological well-being of the general population who had not previously sought mental health support. The programme incorporated a focus on lifestyle medicine and was underpinned by solution focused health coaching. The aim of the paper is to describe the programme according to the Template for Intervention Description and Replication (TIDieR) checklist to provide detailed reporting of the intervention's elements.

**Methods:**

The TIDieR checklist was utilised to comprehensively describe the programme, including theoretical underpinnings, materials, procedures, providers, mode of delivery and tailoring of the programme. The Behaviour Change Technique Taxonomy v2 was used to identify the specific behaviour change techniques used within the solution focused health coaching framework.

**Results:**

The programme was developed to align with the latest evidence-based literature in lifestyle medicine and solution focused coaching. The programme also offered allied health expertise, online educational modules and was tailored to the participants. The programme was delivered online through a telehealth platform.

**Conclusion:**

The TIDieR checklist has enabled the provision of a detailed structure of the EMERALD program intervention. The behaviour change taxonomy has facilitated the outlining of specific techniques used in health coaching sessions. Both structures have operationalised the detail of the intervention for the purposes of replication and informing the literature.

## Introduction

### Rationale for the programme

The rising prevalence of mental health conditions has been associated with significant increases in health and economic burden in high income countries, including Australia. In the last three years, this has been additionally and significantly impacted by the COVID-19 pandemic which has resulted in further increases in mental health challenges both nationwide and globally.^1,2^ Importantly, the COVID-19 pandemic has been linked to an increase in the incidence of mild to moderate mental health concerns, such as anxiety and depression symptoms in individuals who have previously been well^3,4^ and this is comparable to patterns observed in past pandemics.^3,5^ The lockdowns and social distancing also increased the risk of emerging anxiety and depression as well as increased chances of loneliness, a co-morbid factor in both anxiety and depression.^3,6^ Recognising the value of additional services aimed at preventing or alleviating emerging mental health problems during medical pandemics,^7^ there is a need for easily accessible additional services to accommodate the increased burden that arises from population-level impacts such as pandemics.

Prince, Patel^8^ argues that the provision of evidenced based strategies and interventions for the general population can greatly aid in addressing and managing early signs of mental health issues. Further research affirms the importance of intervention and treatment focusing on both physical and mental health within the primary health setting.^9,10^ This approach is integrated into the World Health Organisation Mental health action plan 2023–2033.^11^ Evidence for the effectiveness of lifestyle medicine in alleviating health symptoms in non-clinical populations continues to build,^12^ that is, early intervention lifestyle medicine focused programmes may greatly assist and support the general population who have not previously sought mental health support. Lifestyle medicine has been defined as ‘the application of environmental, behavioural and motivational principles, including self-care and self-management, to the management of lifestyle-related health problems in a clinical setting.’^13^ Lifestyle medicine offers multi-component intervention approaches to support ongoing good mental health as well as early intervention and treatment strategies for common mental health symptoms.^10^ Lifestyle medicine interventions often target behaviours including physical activity, diet, sleep and substance use, with meta-review level evidence demonstrating the large positive impacts of improvements in these behaviours in terms of improvements in well-being and reductions in symptoms of psychological distress.^14^ The use of health coaching offers a facilitated approach which aligns with supporting lifestyle behaviour change, through guidance, support, and accountability.^15^ The EMERging Anxiety, Loneliness, Depression (EMERALD) well-being programme was created to service a gap in the existing mental health services whereby members of the general population, who had previously not sought mental health support but had emerging mild-to-moderate symptoms, could access evidenced based help. By addressing a gap and an increased need made apparent by the COVID-19 pandemic, the EMERALD programme was delivered as a clinical service to help already burdened existing mental health services.

## Aims and objectives

There are few published behaviour change interventions that are described in sufficient detail to allow for replication of an intervention programme.^16,17^ This has been recently noted for health coaching interventions, including a lack of detail on specific behaviour change techniques (BCTs) used and their alignment to health coaching theory.^18^ The aim of this paper is to describe in detail the EMERALD well-being programme using the Template for Intervention Description and Replication (TIDieR) checklist. We describe the theoretical underpinnings and key components of the programme (including a breakdown of each coaching session and associated materials), as well as details on procedures, providers, mode of delivery and tailoring of the programme. This is to assist with standardising the specific ‘ingredients’ for replication and evaluation of the programme, we specify the BCTs adopted as part of the health coaching intervention.^19^

## Methods

### Choice of checklist

The TIDieR checklist was developed by a panel of experts and The Consolidated Standards of Reporting Trials (CONSORT) 2010 to derive a standardised protocol that allows for clearer and precise replication of interventions for replication and implementation.^20^ Similar to Poduval, Ross,^21^ the TIDieR Checklist (Table 1) was selected for this article in order to provide a clear structure to adequately describe the programme intervention, covering who, what, how and when the intervention was implemented. The programme outline has been completed in accordance with the numerical order of the checklist. This approach has been utilised by other authors.^21–24^ Further to the TIDieR checklist, we have included specific telehealth adaptations, in line with the TIDieR-Telehealth checklist^23^ which suggests including the specific telehealth components within the TIDieR 12-item framework.

**Table 1.** Items included in the template for intervention description and replication (TIDieR) checklist: information to include when describing an intervention.

Item number	Item
	Brief name
1.	Provide the name or phrase that describes the intervention
	Why
2.	Describe any rationale, theory or goal of the elements essential to the intervention.
	What
3.	Materials: Describe any physical or informational materials used in the intervention, including those provided to participants or used in intervention delivery or in training of intervention providers. Provide information where the materials can be accessed (e.g. Online Appendix, URL).
4.	Procedures: Describe each of the procedures, activities, and/or processes used in the intervention, including any enabling or support activities.
	Who Provided
5.	For each category of intervention provider (e.g. psychologist, nursing assistant) describe their expertise, background and any specific training given.
	How
6.	Describe the modes of delivery (e.g. face to face or by some other mechanism, such as the Internet or telephone) of the intervention and whether it was provided individually or in a group.
	Where
7.	Describe the type(s) of location(s) where the intervention occurred, including any necessary infrastructure or relevant features.
	When and How Much
8.	Describe the number of times the intervention was delivered and over what period of time including the number of sessions, their schedule, and their duration, intensity or dose.
	Tailoring
9.	If the intervention was planned to be personalised, titrated or adapted, then describe what, why, when and how.
	Modifications
10.	If the intervention was modified during the course of the study, describe the changes, (what, why, when and how).
	How Well
11.	Planned: If intervention adherence or fidelity was assessed, describe how and by whom, and if any strategies were used to maintain or improve fidelity, describe them.
12	Actual: If intervention adherence or fidelity was assessed, describe the extent to which the intervention was delivered as planned.

## Results

### Brief name

The EMERALD programme was an eight-week telehealth multi-component programme designed to support adults to self-manage emerging mental health symptoms, improve their psychological well-being, and increase their ability to adjust to stressors and changes associated with COVID-19. It is unique in the offering of an online (telehealth) health coaching programme utilising early intervention lifestyle medicine focus with the support of health coaching by registered health clinicians as well as further allied health professionals’ consultations (exercise physiologist, dietitian, provisional psychologist, and mental health registered nurse). Additionally, it provided online education resources such as lifestyle medicine modules.

### Why

*Theoretical underpinning.* The EMERALD programme is grounded in the evidence base demonstrating that lifestyle medicine activities have efficacy in ameliorating mental health problems. The EMERALD programme thus focuses on lifestyle factors within its educational content, with lifestyle medicine based health coaching as the relationship format, with online/telehealth operating as the primary method of delivery.

Egger, Binns^13^ describes lifestyle medicine ‘as the application of environmental, behavioural, medical and motivational principles to the management of lifestyle-related health problems in a clinical setting’. Lifestyle medicine has an increasing evidence base for the prevention and treatment of mental health conditions, including as an early intervention for symptoms of depression and anxiety.^14^ Lifestyle medicine is a multimodal framework targeting various modifiable lifestyle factors including physical activity, nutrition, sleep, social connection, and stress management, which are foundational for good physical and psychological health.^25^ Recent clinical trials have demonstrated the effectiveness of lifestyle medicine multimodal interventions for common mental health disorders of anxiety and depression as well as among those who are experiencing subthreshold/clinical symptoms of such disorders. However, this means a core requirement in lifestyle medicine is change of behaviour.^10,14,25,26^

Health coaching is an evidence-based health behaviour change intervention that is derived from behavioural medicine, health and coaching psychology, as well as athletic and performance coaching, to support behaviour change.^27^ There is strong evidence that supports the effectiveness of health coaching which includes focus on lifestyle-based intervention factors as a successful method to improve health outcomes.^28–30^ Health coaching, in particular solution focused coaching, was employed to underpin the EMERALD programme's framework and provide support for behaviour change.^31^ Moore, Jackson^32^ describe four mechanisms of actions that support behaviour change in health coaching intervention: (1) growth promoting relationship, (2) elicit motivation, (3) building confidence and (4) process of change. These mechanisms of action correspond with mechanisms of actions from broader theories of behaviour change.^33,34^

Health coaching has the potential to positively influence behaviour change through delivering personalised guidance, support, accountability, motivation tailored to an individual's distinct needs and goals.^15,27,35^ Findings determined by systematic and integrative reviews which evaluated health coaching report overall effectiveness.^18,36^ However, the techniques across studies are typically not well described and therefore the inability to define the ‘active ingredients’ has led to non-specific results about how health coaching effectively brings about behaviour change.^18,36^ To accurately evaluate an intervention's effectiveness and its ability to be replicated, it is necessary to clearly specify the intervention components.^19^ One way of clearly specifying components or ‘active ingredients’ of an intervention is to use a taxonomy, such as that developed by Michie^19^ which describes 93 BCTs clustered into 16 groups.

**Figure 1.**
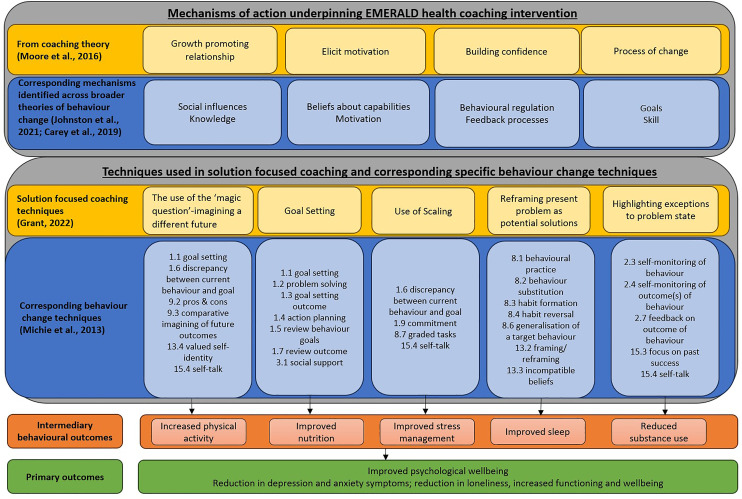
The mechanisms of actions of coaching and their connection to theories of behaviour change; core solution focused coaching techniques used in coaching sessions, and associated behaviour change techniques to support change in lifestyle behaviours and, in-turn, improve psychological well-being.

The BCTs utilised in the EMERALD health coaching sessions and their alignment to the philosophical assumptions of solution focused coaching and techniques are outlined in Figure 1. For example, solution focused health coaching theory suggests that focusing on solutions is one of the key assumptions for behaviour change. In EMERALD, core coaching techniques are used to maintain a focus on solutions throughout sessions, as part of this approach, several specific BCTs are used. For example, the solution focused coaching technique of ‘imagining a different future’, engages the following six BCTs: ‘goal setting’, ‘discrepancy between current behaviour and goal’, ‘pros and cons’, ‘comparative imagining of future outcomes’, ‘valued self-identity’, and ‘self-talk’ (in Figure 1).

This figure outlines how the mechanism of action and behaviour change techniques help facilitate behaviour change to improve lifestyle factors and subsequently psychological well-being. The top layer of the figure depicts the mechanisms of action from coaching theory and the alignment with mechanism of action from broader theories of behaviour change. The second layer depicts the specific solution focused coaching techniques with the corresponding behaviour change techniques, which are numbered according to the Behaviour Change Taxonomy V1.^19^ These techniques are employed together during the health coaching conversation. The techniques/ BCTs promote and support change in the intermediary behavioural outcomes, such as, improvement of physical activity, nutrition, sleep, substance use and stress management. This subsequently leads to the primary outcome of improved psychological well-being.

#### Digital health solution

Prior to COVID-19, there was recognition of the need for eHealth interventions to offer accessible, acceptable interventions for the general population targeting common mental health symptoms.^37^ Telehealth interventions have been shown to produce good health outcomes with common chronic conditions, dietary interventions and behaviour change.^38^ Wind, Rijkeboer^39^ cites that the traditional approach for mental health care did not routinely integrate telehealth sessions, with myths of face-to-face sessions being more important for therapeutic alliance prevailing prior to the COVID-19 pandemic. Deady, Choi^37^ conducted a systematic review of eHealth psychological interventions aimed at preventing depression and anxiety in the general population. The review acknowledged eHealth was in its infancy and found a lack of health prevention trials targeting mental health prevention specifically. Of the eHealth interventions examined, positive effects were found in the short-term, but found inadequate evidence of medium to long term effects. Nonetheless, eHealth technologies have assisted with engagement in treatment while reducing the barriers associated with distance, time and cost.^37,40,41^

### What

*Materials used to deliver the intervention.* The programme was delivered online, using the Health Direct telehealth platform for all appointments in the programme. The online learning modules were delivered via GO 1 learning platform. Participants therefore required access to the Internet, as well as having access to a computer, tablet or smart phone that had a camera and microphone. If participants were not able to access this, telephone calls were offered in lieu of a video call and the modules were sent in an email for printing.

*Information provided to participants (programme content).* Information about the EMERALD programme was advertised via social media, TV, and radio. Links were provided to the EMERALD website which was the first step in the provision of information to interested parties about the contents of the programme www.usc.edu.au/emerald/. The webpage hosted the online registration form as well as contact details of the EMERALD team.

*Synchronous appointments:* There were two types of appointments that offered different content and approaches to the participants, using both health coaching and allied health practitioner (AHP) appointments. The health coaching appointments served as a foundation for the participant journey, while the AHP appointments provided additional and specific expertise in the targeted lifestyle medicine area. Details regarding the timing, frequency and duration of appointments are reported in the ‘Procedure’ section.

The focus of the *health coaching sessions* was to support the self-efficacy of the participant, by targeting the participant selected priority areas of lifestyle medicine. The coaching process focuses on framing the participant to be an expert in their own life and select their areas for behaviour change. The treatment goals were selected by the participant during the coaching process according to their priority areas. To assist with this, a pivotal component used in these sessions was the use of SMART goals, addressing potential barriers and implementing strategies to facilitate the achievement of behaviour change.^42,43^ Throughout the programme, goal setting, goal review, changes in behavioural practice and behaviour substitution were acknowledged, bolstering the participant's self-belief. The next step was to agree on the reviewed goals and plan the participant's targets over the subsequent two weeks. The health coaching appointments were based on a coaching approach of guiding, supporting, and facilitating change through the use of inquiry, active listening and goal setting, with a solution focused approach.^31^ A session-by-session breakdown of content covered and skills used can be accessed in Appendix 1.

*Allied health professional (AHP) sessions* involved clinical assessments to evaluate the participant's health status, clinical guidance to provide tailored recommendations, and advice giving to offer specific health tips and strategies. The primary focus of these sessions was to enhance the participant's skills in managing their health and build their confidence in making changes. AHPs supporting the programme included a dietitian, an exercise physiologist, and a provisional psychologist. The participant's goals formulated in the health coaching session (see above) determined which AHPs they were subsequently booked into and guide the focus of the session e.g. meal planning, or mindfulness practise, etc. The number of appointments with AHPs were flexible, up to a maximum of four. Participants had the option to schedule appointments with different AHPs, or to have multiple appointments with the same AHP.

Specific goals for each type of AHP were as follows. Dietitian: Targeting specific goals regarding reviewing and advising about nutritional intake, offering nutritional advice if required. Exercise Physiologist: Worked with participants to improve their engagement with movement-based and physical activity, offering advice if required. Well-being clinician: The well-being clinician, at the time of this cohort, was a provisional psychologist, offered targeted sessions on skill building and problem solving on mental well-being topics such as psychoeducation of depression, anxiety, stress; sleep hygiene and practices; stress management strategies; building social connection and introducing mindfulness.

*Online modules:* All content was developed based on recent scientific research and evidence-based recommendations. The modules contained education on the importance of the topic, the scientific evidence supporting the topic, practical information to support successful changes and strategies to use and practise regularly. Included were components in written, audio and video formats to accommodate the different learning preferences and modes. Modules (except the Module ‘What is well-being? setting SMART goals’, which was offered to everyone) were selected and released to participants based on their individual goals, for example those who set physical activity goals were sent the Introduction to Physical Activity module. Participants were aware of the complete suite of online learning modules available. Modules were provided to the participant based on a collaborative discussion in health coaching sessions, which would support the existing goals of the participant. The completion time for each module ranged from 15–45 min. At the end of the programme, all completing participants were provided with all learning modules.

**Figure 2.**
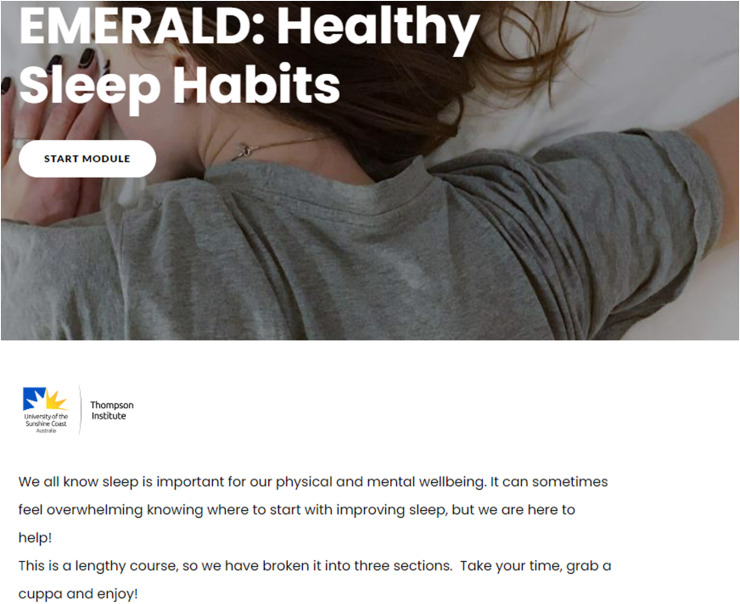
Screenshot of the cover page from the sleep module, EMERALD well-being programme.

The suite of online self-guided learning modules was created and designed to support the participant's knowledge base on each of the areas. These were developed by the clinicians, a research fellow and an educational designer at the Thompson Institute, University of the Sunshine Coast. Modules included:

1. What is well-being? Setting SMART goals.
  An introduction to the importance of well-being and the different lifestyle factors that support well-being and learning about SMART goals. This module was given at the end of the first session to assist reflective practice and consider goals for the next health coaching appointment.
2. What is a healthy diet?
  This module covered the recent scientific evidence about what is important with nutritional food intake.
3. Food safety & kitchen tips and tricks.
  This module covered handy strategies about label reading, understanding ‘best before’ and ‘use by’ dates.
4. Introduction to physical activity.
  This module covered the importance of moving our bodies and how this relates to our mental and physical health.
5. Healthy sleep habits.
  This module covered understanding the role of sleep for our physical and mental health, the different stages of sleep, difference in sleep across various age groups, recommended sleep hygiene and practices to improve sleep (Figure 2).
6. Understanding mental health.
  This module introduced the reader to understanding common signs and symptoms of anxiety, stress and depression and reducing stigma to address some of the barriers.
7. Anxiety management.
  This module examined the physiological and psychological components of anxiety and relating this to neuroscience. Various evidenced based strategies were included to reduce anxiety.
8. Introduction to mindfulness.
  This module introduced the reader to mindfulness, how it works, why this is important and beginning practice of mindfulness of breath.
9. Building social connectedness.
  This module covered the importance of social connection for our mental well-being, the impact of COVID-19 restrictions and lockdowns and offered strategies to improve engagement.
10. Reducing alcohol use.
  This module covered the Australian guidelines for alcohol consumption and the impact of alcohol on our mental health and sleep.
11. Smoking cessation.
  This module covered the role of smoking on our mental and physical health, and offered reflective thinking strategies as well as resources and contact details should one want to stop smoking.

#### Procedures

The online registration process included reading through content outlining the programme objectives, structure, content, and who the programme was intended for. Once the individual navigated this, they then could click a link to registration. This was planned to ensure, as much as possible, each person was fully informed about the programme prior to registration. The initial component of the registration requested consent to the collection of personal information, demographic information, as well as two screening questionnaires to assess the severity of mental health symptoms. These were the Patient Health Questionnaire (PHQ-9) for depression and Generalised Anxiety Disorder Scale (GAD-7) for anxiety.

The PHQ-9 is a 9-item self-report measuring severity of depression in primary care settings. The scale measures the frequency of symptoms in the past two weeks. Each item is scored from 0 (not at all) to 3 (nearly every day). Scores can range from 0–27, with cut off categories for minimal 0–4, mild 5–9, moderate 10–14, moderate-severe 15–19 and severe 20–27. It has demonstrated reliability, convergent/ discriminant validity and responsiveness to change.^44^

The Generalised Anxiety Disorder Scale (GAD-7) is a 7-item self-report measuring severity of anxiety during the past two weeks. Each item is scored from 0 (not at all) to 3 (nearly every day). Total scores can range from 0 to 21, with 0–4 minimal anxiety, 5–9 moderate, 15–21 severe. The measure has been validated for use as a screening tool and severity measure in both primary care and in the general population.^45^

Eligibility into the programme included: (i) 18 years of age or older; (ii) residing in the funded area; and (iii) symptoms ratings on the PHQ-9 and GAD-7 scored below 14, indicating moderate severity of symptoms or below. Exclusion criteria for the programme included: (i) no access to Internet or phone; (ii) not able to speak/ read English; (iii) if they responded ‘yes’ to a question asking if they have seen a mental health professional in the past two years. They were encouraged to contact their previous mental health provider. If an individual scored higher than 1 on the suicidality screening item (item 9 of the PHQ-9), a mental health nurse contacted them to assess risk. If deemed necessary, they were encouraged to seek a review with their GP and provided with information on available support and emergency services. Modification to the screening parameters occurred during the initial stages of the programme. This is discussed in further detail in the modification section.

Once an eligible registration was received, the administrator contacted the participant to arrange their first telehealth session to complete the consent form. After the administrator discussed all items on the consent form, verbal consent was given, the administrator electronically signed the form in front of the participant and emailed them a copy of the electronic consent form; and booked their first health coaching session. The health coach was assigned by the administrator according to coach caseload capacity unless there was a request for a gender specific health coach. Once on the programme, the health coach and participant booked the next health coaching sessions as well as an AHP booking, if this was requested within the subsequent two weeks. Prior to each session, reminder emails were sent. If an appointment was not attended, follow-up contact was attempted by phone and email. Confirmation of the next session emails also sent after every contact.

**Figure 3.**
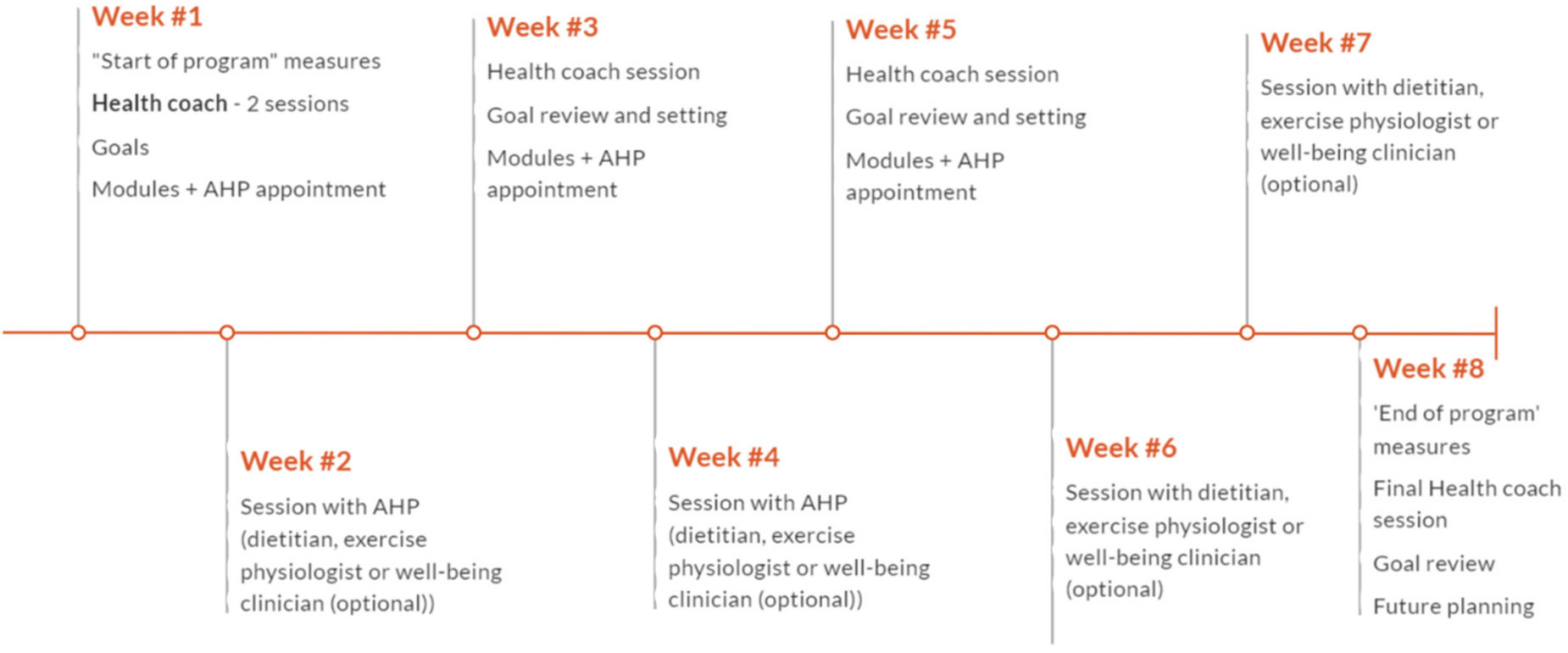
EMERALD programme structure.

The eight-week programme was underpinned by the regular support of an allocated health coach across five telehealth sessions, with the option to see various allied health practitioners up to four appointments in the alternating weeks from the health coach (Figure 3).

This figure demonstrates how the appointments were planned across the eight weeks. Week One: Participant completion of the clinical measures prior to their first session. The first two health coaching sessions, initial to engage and discuss expectation of the programme (see Appendix 1 for breakdown). Second session included goal setting, module selection, and schedule AHP and the next coaching session. Week Two: AHP session, focused on actioning goals and module review. Week Three: Third coaching session review and adjust goals, consider additional online modules and AHP session, book next AHP and coaching session. Week Four: AHP session, action goals and module review. Week Five: Fourth coaching session review and adjust goals, consider additional online modules, two possible AHP sessions, book next AHP and coaching sessions. Week Six and Seven: AHP session, action goals and module review. Week Eight: Participant completion of end-of-programme clinical measures before final coaching session, which reviewed behaviour changes, clinical measure alterations, and consolidation of future plans.

### Who provided

The EMERALD team who delivered the programme consisted of a nurse practitioner, a registered nurse, with 25+ years mental health experience and post graduate qualifications in mental health, an Exercise Physiologist (registered with Exercise Sports Science Australia) with over 10 years experience, a recently graduated Accredited Practising Dietitian, a provisional psychologist completing the Master in Clinical Psychology programme and a research assistant who completed the administration tasks. Some of the clinical staff undertook dual roles of health coach and AHP (exercise physiology, dietetics or well-being). Each of the AHPs were experienced in their fields of practise. The AHP appointments were designed as a typical AHP session, with assessment and clinical advice given, within the scope of a brief intervention/ telehealth format. For any complex or comorbid medical conditions that were not able to be properly assessed within this setting participants were encouraged to seek a review by their general practitioner, with a view for a referral to an external AHP. If a health coach was also required to fill the role for an AHP appointment, these sessions were separately booked to avoid overlap of roles, and the purpose and nature of each appointment type was explained to the participant.

The content development team comprised of the nurse practitioner, registered nurse, research assistant, neuropsychology intern and research fellow. The wider Thompson Institute team, of educational designer, communication and engagement team, partnerships manager and management team supported the marketing, and operations were also involved.

The clinicians were trained in the coaching role by the nurse practitioner, who was an expert in lifestyle interventions for mental health outcomes. The training included the upskilling in specific solution focused techniques (see Figure 1) as well as in general counselling skills such the use of open questions, active listening, and reflections of feeling and content. In preparation for the programme, the training included moderation, direct supervision of coaching skills and structure of the coaching sessions. Throughout the delivery of the programme, the nurse practitioner led clinical supervision to support the ongoing clinical practice.

### How

The programme was delivered entirely online, using the telehealth platform for sessions, email and telephone for booking initial sessions. Appointments throughout the programme were organised at each health coaching session, followed up by email confirmation. All resources were based online, with the option to download a PDF version should they prefer paper-based resources.

### Where

The programme was delivered online and offered to adult residents in Queensland, Australia. The EMERALD team was based at the Thompson Institute, University of the Sunshine Coast, Queensland, Australia. The clinicians provided the telehealth sessions from the Institute or from their home office, depending on lockdown status. The participants were able to access their appointment from a location of their choosing, as long as they had stable internet connection.

#### Telehealth delivery

The programme appointments were offered online using a telehealth video platform due to the impact of the COVID-19 pandemic. The use of telehealth was not traditionally used widely in mental health services prior to the pandemic.^39^ Telehealth service delivery enabled a wider reach of participants without the need to travel and has increased convenience and safety, without the need to increase exposure risk of COVID transmission. As a result the lockdowns, restrictions on travel and physical distancing directives did not impact on the programme deliverability.

### When and how much

Over the course of the eight-week programme, participants were offered five health coaching sessions. These were planned for week one (two sessions), weeks three, five and eight. The initial coaching session, with a duration of 60 min, allowed for time for relationship building, introducing the programme design and content, discussing priority areas, and educating about SMART goals (see Appendix 1 for further details on all health coaching sessions). The middle sessions were scheduled for 30 minutes for goal set up, review and planning. The final session, with a duration of 60 minutes, allowed more time to close their time in the programme. The four optional AHP sessions were offered in weeks two, four, six and seven (see Figure 3). This was purposefully planned to be between health coaching appointments. The sessions were a duration of 45 to 60 minutes, allowing time for assessment, and clinical guidance in the chosen topic.

All sessions were set at a mutually suitable time, and some out of business hours’ time slots were also offered from Monday to Friday.

### Tailoring

The programme adopted a flexible approach to its session content rather than a standardised approach. The content of the health coaching sessions was determined based on the previously set goals and priority areas. A structured agenda was established to maintain a focus on the participant's set goals and prioritised the individual needs during the session. The AHP appointments were tailored according to what the participant required from knowledge deficit, review of strategies or skill building in the particular lifestyle area. During the health coaching session, a participant might identify that they do not possess the knowledge and/or skills they require to complete a goal they have identified. For example, if a person wanted to improve a part of their nutritional intake but have identified that they did not know how, an appointment with the dietitian was considered and booked. This process of separating expert advice helped maintain the fidelity of the health coaching process within session.

In a collaborative relationship, the health coach and participant worked to ensure the targeted behaviour is realistically set according to the person's status, which is generally a small and manageable shift in behaviour, thus creating the next step of behaviour change. Achieving success is crucial as failure can be disheartening, while success fosters confidence and enhances self-efficacy.^46^ An important component of coaching is to use language and goal setting which are positively focused supporting proactive actions rather than removal of a behaviour, i.e. increasing nutritional choices instead of loss of weight.^43,47^ From the initial session, emphasis was given to encouraging the idea that participants are their own experts of the own experiences. The coach's role was to provide support and guidance, helping the participants to discover their solutions and work on goals tailored to their current position and the subsequent steps they would take.

If there were issues with Internet connectivity or participants were not comfortable using video call, telephone sessions were offered. If participants were not able to access the online modules, printed modules were sent in the mail.

### Modifications

When the programme first launched, the aim was for the programme to be eligible to those who scored in the mild range (<9) of the screening tools, PHQ-9 and GAD-7. Those who were screened higher (>9) were not offered to be contacted and further screened, but given information and resources, as well as encouraged to seek a review by their General Practitioner.

However, after the initial launch, it was promptly observed that many people applying for the programme were scoring up to the moderate ranges of either or both scales (<14). This observation was discussed in a multidisciplinary team meeting and the eligibility was adjusted to moderate levels of symptoms (<14). The screening was further modified to offer a clinical screening call to the people who scored higher than <14, for the purposes of assessing suitability. The person could accept or decline this offer on the online registration. Those scoring above moderate level of severity on the PHQ-9 and/or the GAD-7 were contacted and assessed by a mental health nurse. If it was determined through the conversation that the depression and/or anxiety symptoms were markedly impacting their functioning and/or the clinician identified the support required was outside the scope of the EMERALD programme, they were advised to consult their General Practitioner for further assessment and referral to a mental health clinician for intensive mental health treatment and support. The same modification was applied, part way through the programme, for individuals who indicated that they had consulted a mental health professional in the past two years. These individuals were offered a call with the EMERALD lead clinician or mental health nurse to discuss the content and purpose of the EMERALD programme. A clinical judgement was then made to determine whether Lifestyle Medicine would provide an adequate level of support for the individual's needs, or whether a higher level of support beyond the scope of the EMERALD programme was required (Supplemental material).

### How well

The nature and size of the intervention meant a pragmatic approach to staffing was required. The number of staff on the team was small and just one of each discipline (dietitian, exercise physiologist, provisional psychologist and a mental health nurse) so there was necessity for some staff to undertake dual roles undertaking both health coaching sessions and the AHP sessions. In the programme, there was a clear demarcation of the roles and content between the health coaching sessions and the AHP sessions. To assist with the separation of these distinct roles the programme lead ensured training and supervision focused on the fidelity of the coaching model and use of coaching appointment templates for each session.

To maintain the fidelity of the health coaching framework throughout the intervention, the programme lead (Nurse Practitioner), trained each team member using solution focused health coaching techniques and methodologies. This incorporated didactic teaching of the framework, engaging in role plays, simulating with staff as participants prior to the launch of the programme. During the programme ongoing quality assurance of the health coaching sessions was conducted by seeking participant permission for the programme lead to join appointments as a silent observer and provide constructive feedback to the health coach after the appointment. The nurse practitioner also led fortnightly group clinical supervision to support adherence to the health coaching role using BCTs, as well as discussing individual presentations. Group supervision offered didactic teaching, reflective practice and peer feedback. Each AHP attended regular monthly specialist supervision for their allied health roles.

To assess the effectiveness of the programme, online clinical questionnaires were completed at the start of the programme (within the week prior to commencing the programme), and upon its completion, within the week prior to the final health coaching session. The ‘end of programme’ measures were purposely captured prior to the last session in order to discuss changes to the scores with the participant in their final review. This enabled discussion about possible changes to their mental health symptoms, functioning, loneliness and well-being in the previous eight weeks. This allowed us to reduce the administrative burden on participants with task completion that did not serve them an individual benefit.

The programme being entirely online allowed participants across a large geographical area to access the programme despite the distance. The numerous lockdowns did not disrupt programme delivery.

## Discussion

This paper utilises the TIDieR checklist framework, including the TIDieR-Telehealth components, to comprehensively outline the EMERALD well-being programme, facilitating a standardised description of its various components, including theoretical framework, materials, procedures, providers, delivery mode, structure, dosage, customisation, and adaptations. The programme's inception was prompted by the anticipated rise in mental health challenges due to the COVID-19 pandemic. Programme development began in March 2020. In August 2020 the programme was launched. The first cohort of the programme ran from August 2020 until June 2021. Since then, under different funding, the EMERALD programme continued to operate until March 2024.

The EMERALD well-being programme stands out for its integrated approach encompassing early intervention, solution focused health coaching, lifestyle medicine, telehealth delivery, online resources, and a multidisciplinary team of registered health professionals to target mental health well-being outcomes. Currently, research literature lacks detailed descriptions of BCTs utilised within health coaching roles, hindering effective replication. By detailing the programme's protocol, this paper enables clinicians and researchers to replicate the intervention, fostering further investigation. Furthermore, the use of the TIDieR checklist enabled the description of the responsive changes to the programme. The outcomes of the EMERALD programme will be analysed and discussed in a future paper.

## Conclusion

In this paper, our endeavour has been to provide a comprehensive outline of the EMERALD well-being programme using the TIDieR checklist to describe the programme, including theoretical underpinnings, materials, procedures, providers, mode of delivery and tailoring of the programme. Currently in the literature, there is a scarcity of information outlining BCTs within a coaching framework that can allow for the evaluation and replication of a mental well-being programme. By utilising the TIDieR checklist in conjunction with the BCT taxonomy (v1) to identify the specific BCTs, the aim is to equip researchers and clinicians with the necessary detail for replication and progression of similar online mental health focussed interventions.

The TIDieR checklist is becoming increasingly utilised to describe multi-component interventions for evaluation and replication.^48–51^ A strength of using the TIDieR framework is its structural outline, which aids documentation by providing a comprehensive breakdown of each component in the programme. Although the framework was applied retrospectively, the importance of detailing the components of an intervention during its development is acknowledged.

## Supplemental Material

sj-docx-1-dhj-10.1177_20552076241288381 - Supplemental material for ‘EMERALD’ online early intervention programme for psychological well-being: A detailed description using the TIDieR checklist Supplemental material, sj-docx-1-dhj-10.1177_20552076241288381 for ‘EMERALD’ online early intervention programme for psychological well-being: A detailed description using the TIDieR checklist by Monique Jones, Alexandra P Metse, Andrew Watkins, Daniel F Hermens and Christina Driver in DIGITAL HEALTH

## Appendix 1

### Brief breakdown of structure of each health coaching session

#### Health coach session 1

The initial session encompassed introductions between the clinician and participant, focusing on establishing rapport and fostering a therapeutic relationship^52^; incorporated into the session was a discussion about the programme's design, which then transitioned into exploration of the participant's current healthy behaviours in lifestyle areas. Through dialogue with the health coach, the participant described their then-current lifestyle habits and routines, how they were currently supporting a healthy lifestyle, they identified barriers to engaging in healthy practices, and expressed the areas they aimed to work on during the programme. After the end of the first session, participants were provided with a reflective module. This module covered the topics of what is well-being, why well-being is important and briefly outlined each of the areas of the programme that the participant could focus on. The introduction to using SMARTER goals was included in the module in preparation for the second session where the participants identify the areas they view as priority for them to work on. The use of identifying priority areas chosen by the participant then enabled the creation of tailored SMARTER goals which the participant would engage in between appointments. The SMARTER acronym refers to Specific, Measurable, Achievable, Realistic, Timely, Evaluate, Review to ensure a targeted approach to actionable behaviours. The framework SMART goals is a widely used framework for practice of goal pursuit.^43^ Goals were reviewed and readjusted as necessary.

#### Health coach session 2

This session encompassed a discussion of priority areas, involving the ranking of these areas based on their importance. It also involved identifying an area that wouldbe the easiest to make immediate changes, aiming to support confidence building and create a sense of achievement. Goal setting was initiated applying the SMARTER goal acronym to facilitate support targeted, achievable goals. Commonly the key areas of achievability and realism was worked through with the participant to set parameters where they were 80% confident that they could achieve this in the two weeks prior to their next health coaching appointment. If they were not that confident, the goal was adjusted in some way to achieve 80% confidence rating.^53^ Identifying potential barriers that could come up and developing strategies that could assist reducing the possible impact was also an important piece of the conversation.^53^ The barriers identified may have previously impeded progress, making it crucial to address and explore ways to diminish these barriers through strategies. When discussing resource support in relation to the participant's goals, this included which modules could be beneficial along with evaluating the value of an AHP appointment that would support their progress on a particular goal. For example, for a physical activity goal, the Introduction to Physical Activity module was released and the participant was booked in to speak with the Exercise Physiologist, if it was thought a more specialised conversation would be helpful to them. These appointments were booked in, the modules released to them, and the next health coaching session was also scheduled.

#### Health coach sessions 3 & 4

The subsequent sessions were centred around reviewing goals, with participants rating the degree of achievement (using the scale 1–10, where 10 indicates complete achievement and 1 signifies no initiation); conducting a thorough evaluation and review of goals was critical for fostering reflective thinking, highlighting successes of the goals, identifying barriers and challenges, and understanding the impact on goal achievement. This not only facilitated the establishment of readjusted goals but also provided an opportunity for the participant to reflect and gain feedback from this learning.^54^ This prompted the discussion about which resource support (modules/ AHP appointments) would be beneficial within the next fortnight. Consideration was given to whether the participant envisioned potential obstacles to pursuing these goals and identified strategies to overcome them.

Throughout the goal setting process, the participant determined the number of goals, with the health coach providing support by assessing capacity and achievability of the targeted number of goals. The collaborative approach facilitated the growth of the participant.^43^ The next appointments were booked at the end of each health coaching session.

#### Health coach session 5

The aim of final session was to engage in reflective practice^43^ and review the behaviour changes made in the programme. This was intended to acknowledge and celebrate the achieved goals, recognising the factors that contributed to their success, and identifying the goals and supports crucial for sustaining momentum moving forward. Additionally, the results of the clinical scales at the end of the programme were discussed. Comparing the scores between the ‘start of programme’ and ‘end of programme’ scores was helpful to recognise gains made that has impacted on functioning, perception of well-being, sense of loneliness and symptoms of anxiety and depression. Any unchanged areas were also discussed and acknowledged. Relapse prevention (RP) is a strategy that is used in recurrent mental health disorders, such as depression and addiction.^55^ RP has the approach that acknowledges fluctuation in mood, behaviours, recognising old habits, and feelings can emerge. Without consideration to these times, participants may engage in old habitual ways. This was discussed and this included the acknowledgement and understanding of fluctuations in progress of behaviour change; the impact of stressors and the vulnerability that can emerge in the moments to return back to old behaviours; returning to the healthier health behaviours using goal setting, making achievable steps back to their desired outcome. Discussing RP has been shown to reduce the risk of relapse.^56^ The conversation also included the importance for self-compassion as the participants continue with behaviour changes. Taking a compassionate perspective has been positively associated with good mental health and greater likelihood of engagement in healthy behaviours.^57,58^
