# Supplementary material for: ‘EMERALD’ online early intervention programme for psychological well-being: A detailed description using the TIDieR checklist
Source: Digit Health. 2024 Oct 15;10:20552076241288381. doi: 10.1177/20552076241288381 (PMC11483705; doi:10.1177/20552076241288381)
Supplement: sj-docx-1-dhj-10.1177_20552076241288381 - Supplemental material for ‘EMERALD’ online early intervention programme for psychological well-being: A detailed description using the TIDieR checklist [file sj-docx-1-dhj-10.1177_20552076241288381.docx]

**Supplementary Material**

**Generalised Anxiety Disorder Scale (GAD-7)**

| Over the last **two weeks**, how often have you been bothered by the following problems? | **Not at all** | **Several days** | **More than half the days** | **Nearly every day** |
| --- | --- | --- | --- | --- |
| 1. Feeling nervous, anxious, or on edge | 0 | 1 | 2 | 3 |
| 2. Not being able to stop or control worrying | 0 | 1 | 2 | 3 |
| 3. Worrying too much about different things | 0 | 1 | 2 | 3 |
| 4. Trouble relaxing | 0 | 1 | 2 | 3 |
| 5. Being so restless that it is hard to sit still | 0 | 1 | 2 | 3 |
| 6. Becoming easily annoyed or irritable | 0 | 1 | 2 | 3 |
| 7. Feeling afraid, as if something awful  might happen | 0 | 1 | 2 | 3 |

**Scoring GAD-7 Severity**

This is calculated by assigning scores of 0, 1, 2, and 3 to the response categories, respectively, of “not at all,” “several days,” “more than half the days,” and “nearly every day.”

GAD-7 total score for the seven items ranges from 0 to 21.

0–4: Minimal

5–9: Mild

10–14: Moderate

15–21: Severe

Reference: Williams, N. (2014). The GAD-7 questionnaire. Occupational Medicine, 64(3), 224-224. https://doi.org/10.1093/occmed/kqt161 %J Occupational Medicine

**Patient Health Questionnaire (PHQ-9)**

| Over the **last two weeks**, how often have you been bothered by any of the following problems? | **Not at all** | **Several Days** | **More than half the days** | **Nearly every day** |
| --- | --- | --- | --- | --- |
| 1. Little interest or pleasure in doing things | 0 | 1 | 2 | 3 |
| 2. Feeling down, depressed, or hopeless | 0 | 1 | 2 | 3 |
| 3. Trouble falling or staying asleep, or sleeping too much | 0 | 1 | 2 | 3 |
| 4. Feeling tired or having little energy | 0 | 1 | 2 | 3 |
| 5. Poor appetite or overeating | 0 | 1 | 2 | 3 |
| 6. Feeling bad about yourself — or that you are a failure or have let yourself or your family down | 0 | 1 | 2 | 3 |
| 7. Trouble concentrating on things, such as reading the newspaper or watching television | 0 | 1 | 2 | 3 |
| 8. Moving or speaking so slowly that other people could have noticed? Or the opposite — being so fidgety or restless that you have been moving around a lot more than usual | 0 | 1 | 2 | 3 |
| 9. Thoughts that you would be better off dead or of hurting yourself in some way | 0 | 1 | 2 | 3 |

**Scoring PHQ-9 Severity:** 
0-4: Minimal
5-9: Mild
10-14: Moderate
15-19: Moderate severe
20-27: Severe

Reference: Cameron, I. M., Crawford, J. R., Lawton, K., & Reid, I. C. (2008). Psychometric comparison of PHQ-9 and HADS for measuring depression severity in primary care. *Br J Gen Pract*, *58*(546), 32-36. https://doi.org/10.3399/bjgp08X263794
